# Magnetic resonance imaging subtraction vs. pre- and post-contrast 3D gradient recalled echo fat suppressed imaging for evaluation of the canine and feline brain

**DOI:** 10.3389/fvets.2024.1346617

**Published:** 2024-01-23

**Authors:** Heather Simon, Silke Hecht, Constance Fazio, Xiaocun Sun

**Affiliations:** ^1^Department of Small Animal Clinical Sciences, University of Tennessee College of Veterinary Medicine, Knoxville, TN, United States; ^2^Office of Information Technology, University of Tennessee, Knoxville, TN, United States

**Keywords:** MRI, contrast medium, encephalopathy, dog, cat, central nervous system

## Abstract

Subtraction magnetic resonance imaging (MRI) has been reported to increase accuracy in the diagnosis of meningeal and inflammatory brain diseases in small animals. 3D T1W gradient recalled echo (GRE) techniques have been proposed as a suitable alternative to conventional spin echo sequences in imaging the canine brain. The aim of this study was to compare subtraction images and paired pre- and post-contrast 3D T1W GRE fat suppressed (FS) images in canine and feline MRI studies using clinical diagnosis as the gold standard. Paired pre- and post-contrast T1W 3D FS GRE images and individual subtraction images of 100 small animal patients were randomized and independently evaluated by 2 blinded observers. Diagnosis categories were “normal,” “inflammatory,” “neoplastic,” and “other.” Clinical diagnosis was made in the same categories and served as the gold standard. Image interpretation results were compared to the clinical diagnosis. Interobserver agreement was determined. Clinically, 41 studies were categorized as “normal,” 18 as “inflammatory,” 28 as “neoplastic,” and 13 as “other.” The agreement of the pre- and post-contrast GRE images with the gold standard was significantly higher than that of the subtraction images (*k* = 0.7491 vs. *k* = 0.5924; *p* = 0.0075). The largest sources of error were misinterpretation of “other” as “normal” and “normal” as “inflammatory.” There was no significant difference between the two observers (*p* = 0.8820). Based on this study, subtraction images do not provide an advantage to paired pre- and post-contrast FS GRE images when evaluating the canine and feline brain.

## 1 Introduction

Due to the difficulty of obtaining histopathologic samples for the antemortem diagnosis of canine and feline brain disease, clinicians frequently combine signalment, clinical signs, advanced imaging findings, cerebrospinal fluid analysis results, and treatment response to formulate differential diagnoses and treatment plans. Heavy reliance is placed on non-invasive measures such as magnetic resonance imaging (MRI) to obtain presumptive diagnoses. Previous studies have shown that MRI is highly sensitive and specific for inflammatory and neoplastic brain lesions in dogs and has high interobserver agreement (1–3).

Subtraction is a post processing procedure performed after MR images have been acquired and involves one image being digitally subtracted from another. This can be done for follow-up studies (e.g., subtracting images obtained at different points in time to assess for disease progression), or during the same scan (e.g., for improved visualization of contrast enhancing structures by subtracting pre- from post-contrast images). Most studies in subtraction techniques are reported in human neuroimaging. Applications include detection of multiple sclerosis plaques and evaluation of changes over time (4–7), evaluation of tumor growth in gliomas and prediction of cleavage planes in surgical resection of meningiomas (8, 9), detection of amyloid-related imaging abnormalities with edema in patients with Alzheimer’s disease (10), evaluation of subtle changes in the brains of human infants and children (11), improved delineation of contrast-enhancing tumors adjacent to hemorrhagic lesions (12, 13), improved sensitivity for the detection of ischemic lesions in post-surgical cardiac patients undergoing brain MRI (14), and improved identification of subtle meningeal disease in people (15–17). Added advantages of using subtraction images may include a decrease in image reviewing time (4, 14), and a reduction of intravenous contrast medium dosages in people (18).

Studies on using subtraction techniques in veterinary MRI are limited to date. One study concluded that subtraction images increase the conspicuity of normal canine meninges (19). One case report describes subtraction images being used to differentiate chondroma and squamous cell carcinoma in two beagle dogs (20). Another study reports improved detection of meningeal, articular and muscular contrast enhancement in the spine of dogs afflicted with steroid responsive meningitis arteritis (21). Inflammatory brain diseases in dogs can have variable imaging manifestations including subtle meningeal changes. Granulomatous meningoencephalitis specifically has been likened to multiple sclerosis in people in its leptomeningeal pathophysiology (22). While subtraction images have proven helpful in people with meningeal lesions secondary to multiple sclerosis, reports in canine patients with inflammatory encephalopathies are conflicting. One study found that subtraction images were not superior to T1-weighted post contrast images and that, overall, MRI was poor at detecting meningeal pathology (23). A subsequent study compared subtraction images vs. pre- and post-gadolinium T1-weighted spin echo (SE) image pairs in dogs with intracranial inflammatory conditions. This study concluded that the subtraction images performed better than the pre- and post-contrast imaging pairs, primarily due to their ability to detect intra-axial lesions (24).

Three-dimensional T1-weighted gradient recalled echo (3D T1W GRE) sequences with fat suppression have recently gained popularity as an alternative to standard SE sequences and other techniques (25). Fat suppression alone can increase subtle lesion detection. In a study on meningeal enhancement in dogs, chemical fat suppression significantly increased detection of meningeal enhancement and was associated with the highest inter-observer agreement (26). An added advantage of 3D GRE sequences is their thin slice thickness and lack of an interslice gap. In one study, the 3D T1W GRE images (1 mm slice thickness) had superior anatomic detail when evaluating canine patients for facial nerve pathology compared to conventional T1W SE images (3–3.5 mm slice thickness) (27). In a human study, the same 3D T1W GRE technique, which does not have an inversion recovery pulse, had superior performance in detecting brain tumor enhancement when compared to the more traditional post-contrast inversion recovery fast GRE sequence (28). A canine study found that a 3D T1W GRE sequence is a suitable alternative to the more traditional two-dimensional T1W SE sequence for brain MRI (29).

The aim of this study was to compare subtraction images and paired pre- and post-contrast 3D T1W GRE fat suppressed (FS) images to the gold standard of clinical diagnosis in 100 canine and feline MRI studies. The secondary purpose was to assess inter-observer agreement for both techniques. The hypothesis was that there would be no difference in diagnostic yield and interobserver variability between subtraction images and paired pre- and post-contrast 3D T1W FS GRE images.

## 2 Materials and methods

### 2.1 Patients

A retrospective study was performed at The University of Tennessee College of Veterinary Medicine on 100 canine and feline patients receiving a brain MRI study. Medical records were reviewed by the first author (HS), a senior resident in veterinary neurology/neurosurgery. Medical record data recorded were age, gender, breed, weight, clinical history, clinical and neurologic examination findings, cerebrospinal fluid (CSF) analysis results, ancillary test findings, histopathology (if available), treatment response, and follow-up. All patients had current (within 1 month) blood analysis including complete blood count and serum chemistry and a complete neurologic exam within 24 h of imaging. Patients may or may not have had thoracic radiographs, cerebrospinal fluid (CSF) analysis, infectious disease titers, histopathology, or other additional testing. Inclusion criterion was availability of pre- and post-contrast T1W 3D FS GRE sequences and dynamic subtraction images of diagnostic quality. Diagnosis codes were designated for patients by the first author, using all available information (medical record data, clinical history, physical and neurologic examination findings, cerebrospinal fluid analysis results, ancillary test findings, imaging findings, treatment response, follow-up, and histopathology). Diagnosis codes were divided into four groups: 0 for normal, 1 for inflammatory disease, 2 for neoplasia, and 3 for other (any disease entity outside of these categories such as vascular, congenital, toxic/metabolic, etc.). The category of inflammatory disease included infectious and non-infectious causes. The neoplastic category included primary and metastatic disease.

### 2.2 Imaging protocol

MRI was performed using a 1.5 T MRI system (MAGNETOM Espree TM, Siemens Medical Solutions, Malvern, PA). All patients were anesthetized and positioned in dorsal recumbency. MRI protocols were tailored to individual patients and typically included the following sequences: Sagittal T2W SE; transverse T1W SE, T2W SE, T2*W GRE, T2W 3D TSE with variable flip angle (Sampling Perfection with Application optimized Contrasts using different flip angle Evolution; “SPACE”), T2W Fluid Attenuated Inversion Recovery (FLAIR), Proton Density-W SE, and diffusion weighted imaging (DWI) with ADC map; transverse pre- and post-contrast 3D T1W gradient recalled echo (GRE) sequence with fat suppression (Volume Interpolated Body/Breath hold Examination; “VIBE”) and subtraction; post contrast sagittal and transverse T1W SE; and post contrast dorsal T1W SE with fat saturation. Pre- and post-contrast 3D T1W GRE FS sequences were acquired with 1 mm slice thickness. Contrast medium was administered intravenously at 0.1 mmol/kg (Omniscan TM; Gadodiamide, GE Healthcare Inc., Marlborough, MA 01752, United States).

### 2.3 Image evaluation

Pre- and post-contrast T1W 3D FS GRE images and subtraction images were separately randomized and anonymized by the first author (HS) and were provided to each reviewer blinded to other imaging sequences and the clinical information. Two experienced board-certified veterinary radiologists (SH, CF) were separately provided with the subtraction images and the pre- and post-contrast T1W 3D FS GRE image pairs and asked to designate a diagnosis code of 0, 1, 2, or 3 (same categories as above) for each scan.

### 2.4 Statistical analysis

The statistical analysis was performed by a university employed statistician (XS). Chi square test and kappa coefficient test were performed to determine agreement between the subtraction images and the paired pre- and post-contrast T1W 3D FS GRE images, respectively, with the clinical diagnosis. Kappa statistics were also used to determine interobserver agreement. Statistical significance was set at *p* < 0.05. Analyses were conducted in SAS 9.4 TS1M6 for Windows 64× (SAS institute Inc., Cary, NC).

## 3 Results

To achieve the target of 100 patients to be included in the study, 117 MRI studies were reviewed. Seventeen cases were excluded due to inadequate quality of the subtraction images due to misregistration error (change in patient position between pre- and post-contrast images).

There were 9 cats and 91 dogs. Overall, there were 51 females (45 neutered) and 49 males (34 neutered). The cats were all domestic short hair. The canine breeds included were mixed breed dog (*n* = 26), Chihuahua (*n* = 5), Golden Retriever (*n* = 4), Boxer (*n* = 4), Corgi (*n* = 3), Labrador Retriever (*n* = 3), Australian Shepherd (*n* = 3), Shih Tzu (*n* = 3), Dachshund (*n* = 3), Pug (*n* = 3), English Bull Dog (*n* = 2), Yorkshire Terrier (*n* = 2), Cavalier King Charles Spaniel (*n* = 2), Havanese (*n* = 2), Boston Terrier (*n* = 2), Miniature Schnauzer (*n* = 2), and one each of the following: Australian Kelpie, Bassett Hound, Belgian Malinois, Blue Tick Hound, Border Collie, Cairn Terrier, Australian Cattle Dog, Cocker Spaniel, Coonhound, Dalmatian, French Bulldog, German Shepherd Dog, Otterhound, Maltese, Miniature Pinscher, Pitbull, Pomeranian, Rat Terrier, Rhodesian Ridgeback, German Shorthair Pointer, Staffordshire Terrier and Toy Poodle. Ages ranged from 3 months to 13 years.

Clinical diagnosis codes (gold standard) were as follows: 41/100 normal, 18/100 inflammatory, 28/100 neoplasia, and 13/100 other.

Of the patients in category “normal,” the diagnoses were as follows: idiopathic/cryptogenic epilepsy (*n* = 20), idiopathic vestibular disease and/or facial nerve paralysis (*n* = 8), presumed primary cardiac or syncopal episodes (*n* = 4), primary behavioral abnormalities (*n* = 2), otitis media (*n* = 2), and one case each of primary ocular disease, canine cognitive dysfunction, tooth root abscess, portosystemic shunt, and intermittent apnea.

Of the patients in category “inflammatory,” the diagnoses were as follows: meningoencephalitis of unknown etiology (*n* = 15), focal meningitis secondary to extension of otitis media/interna (*n* = 2), and idiopathic cerebellitis (*n* = 1).

Of the patients in category “neoplasia,” the diagnoses were as follows: extra-axial tumor/presumptive meningioma (*n* = 13), intra-axial tumor/presumptive glioma (*n* = 8), aggressive nasal mass (*n* = 2), pituitary mass (*n* = 2), and one case each of choroid plexus tumor, trigeminal nerve sheath tumor, and infiltrative mass along the carotid artery.

Of the patients in category “other,” the diagnoses were as follows: cerebrovascular accidents (*n* = 5), hydrocephalus (*n* = 3), cerebellar hypoplasia (*n* = 2), and one case each of metabolic/toxic bilaterally symmetric brain lesions, multiple skull fractures, and supracollicular fluid accumulation.

The agreement of the pre- and post-contrast T1W GRE images with the gold standard was significantly higher than that of the subtraction images (*k* = 0.7491 vs. *k* = 0.5924; *p* = 0.0075).

**Figure 1:**
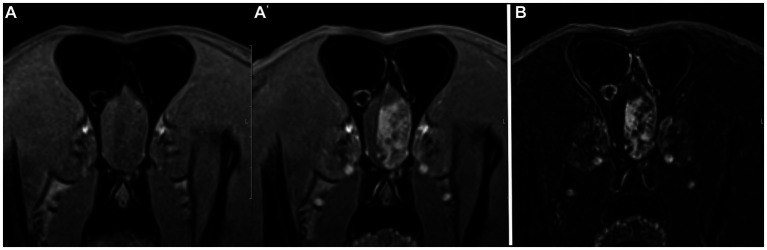
Example of good agreement of both the pre- and post-contrast T1W 3D FS GRE images **(A,A’)** and the subtraction image **(B)** with the gold standard. This patient was diagnosed with an extra-axial brain tumor (meningioma, presumptive). Both reviewers made the correct diagnosis of “neoplasia” on both image sets. Note the large heterogeneously contrast enhancing mass in the left rostral cranial vault.

**Figure 2:**
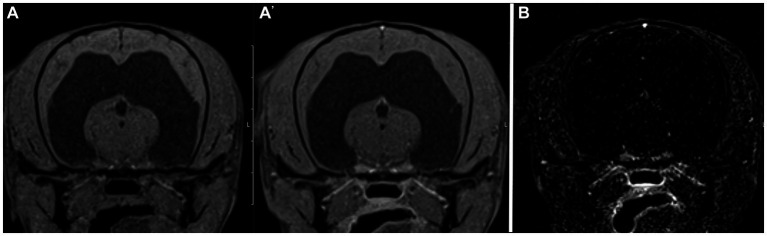
Example of good agreement of the pre- and post- contrast T1W 3D FS GRE images **(A,A’)** but poor agreement of the subtraction image **(B)** with the gold standard. The pre- and post-contrast GRE images were correctly interpreted as “other” by both reviewers, while a false positive diagnosis of “normal” was made based on the subtraction image. This patient was diagnosed with a hydrocephalus, likely congenital.

**Figure 3:**
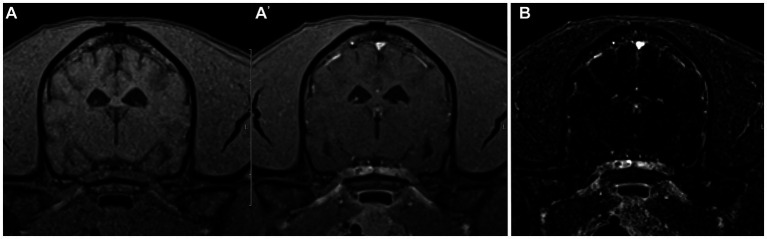
Example of good agreement of the pre- and post-contrast T1W 3D FS GRE images **(A,A’)** but poor agreement of the subtraction image **(B)** with the gold standard. The pre- and post-contrast GE images were correctly interpreted as “normal” by both reviewers, while a false positive diagnosis of “inflammatory” was made based on the subtraction image which has evidence of meningeal contrast enhancement. This patient was diagnosed with idiopathic vestibular disease.

**Figure 4:**
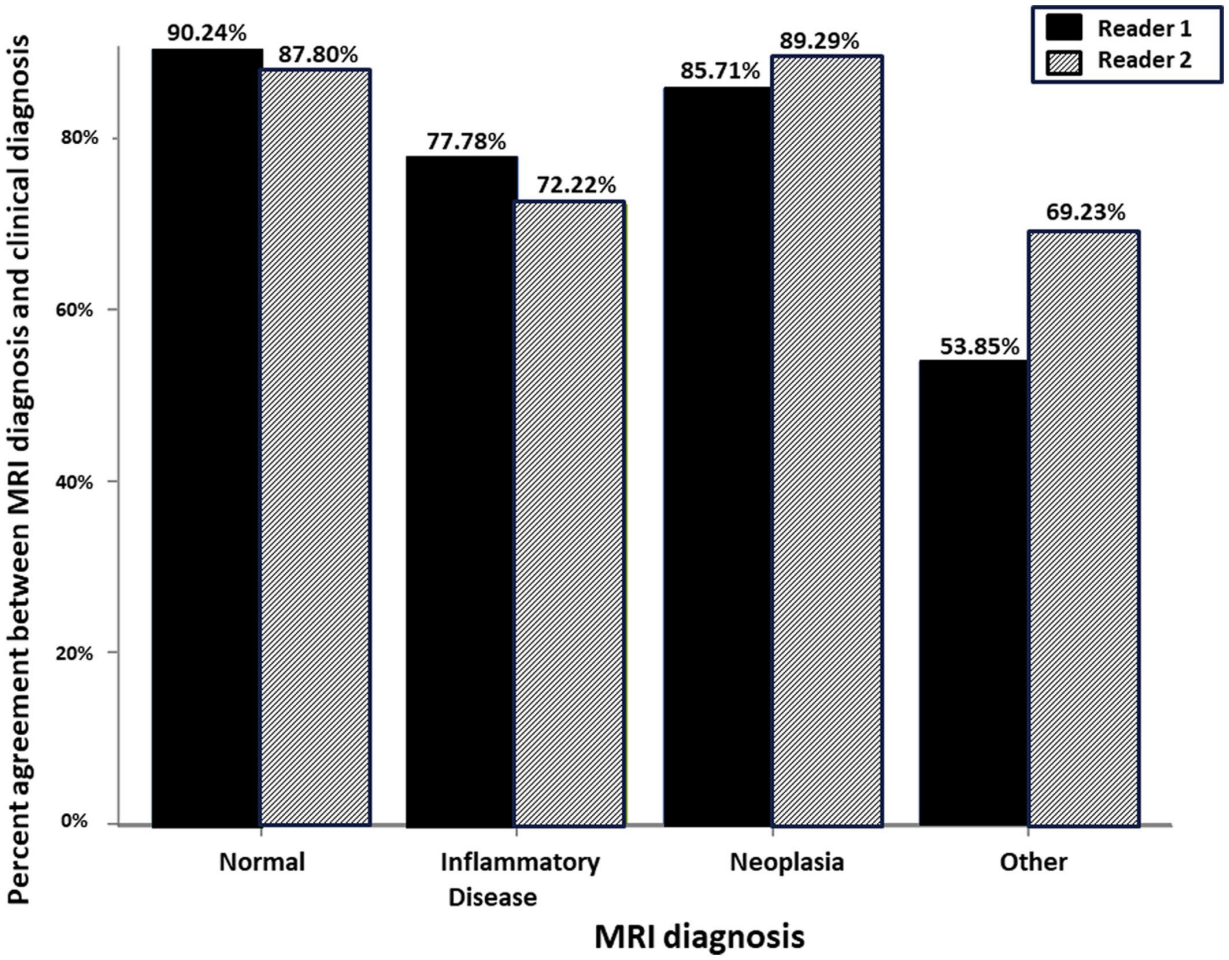
Schematic representation of the percent agreement between the MRI Diagnosis based on paired pre- and post-contrast T1W FS GRE and the gold standard clinical diagnosis. There was no significant difference between the two readers.

**Figure 5:**
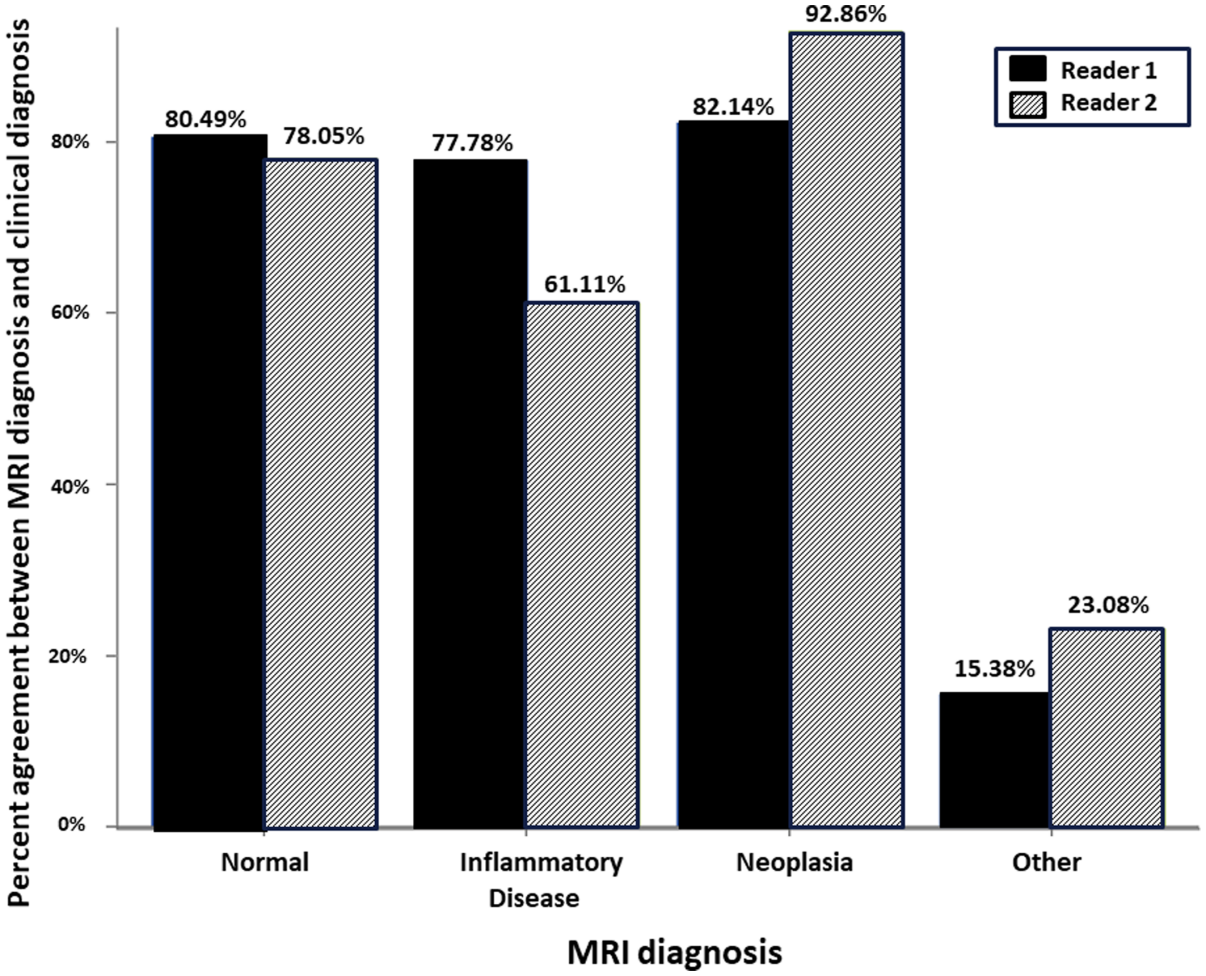
Schematic representation of the percent agreement between the MRI Diagnosis based on subtraction MR image and the gold standard clinical diagnosis. There was no significant difference between the two readers.

There was similar agreement between the pre- and post-contrast T1W GRE and the subtraction images with the clinical diagnosis in patients with intracranial neoplasia (88% vs. 88%; Figure 1). The largest discrepancies between agreement of pre- and post-contrast T1W GRE and subtraction images with the clinical diagnosis were misinterpretation of “other” as “normal” (65% vs. 23%; Figure 2) and “normal” as “inflammatory” (17% vs. 7%; Figure 3). There was no significant difference between the two observers (*p* = 0.8820). Details of the individual interpretation by the two readers can be found in Figures 4, 5 and Supplementary Figures S1–S4.

## 4 Discussion

With subtraction imaging, tissues that remain static between successive scans will null out, while tissues that have changed will be highlighted. When using subtraction for pre- and post-contrast images, contrast-enhancing tissues will be accentuated. Based on this study, MRI subtraction images did not provide an advantage to paired pre- and post-contrast GRE images when evaluating the canine and feline brain.

One major source of discrepancy between subtraction images and the gold standard was misinterpretation of “normal” studies as “inflammatory.” This may be attributable to normal meningeal contrast enhancement being mistaken for pathology. It has been reported that the degree of meningeal enhancement in subtraction images of normal canine brains is greater than expected, and that some degree of enhancement in the pachymeninges and leptomeninges is physiologic due to their lack of a blood-brain-barrier (19). Another less likely explanation may have been instances of false positive identification of intra-axial lesions. 12% of dogs with idiopathic epilepsy had evidence of intra-axial enhancing lesions on subtraction images in a prior study, attributed to either false positive lesions or possibly representing true pathology (24). A third possibility for false positive results with subtraction images is artifact secondary to large slice thickness (10). However, we feel that this is less likely since our images were created with thin slices and no interslice gap. Finally, even though both radiologists involved in image interpretation in this study have experience in neuroimaging, they did not have enhanced training at interpretation of subtraction images which may have affected the results.

The other major source of error was misinterpretation of “other” as “normal.” This can easily be explained by “other” lesions often being static/non-enhancing between scans (e.g., skull defects, intracranial fluid accumulations) and thus being nulled on subtraction images. While it is unlikely that these lesions would be missed in clinical practice where other sequences (e.g., T2-weighted images) are acquired in the frame of a complete brain MRI examination, it is important to recognize the limitations of subtraction images to identify non-contrast-enhancing lesions.

The major limitation of our study is the lack of a definitive diagnosis (histopathology) in most cases. Brain biopsies are not frequently performed in veterinary medicine due to their invasiveness, cost, and potential need for special navigational equipment. The diagnoses in our patient cohort were based on a combination of signalment, history, neurologic exam findings, imaging findings, results of further tests, and follow-up, similar to the methodology used in other studies (30, 31). MRI alone has a sensitivity and specificity of 94.4 and 95.5%, respectively, for the diagnosis of brain disease in dogs (1). The diagnosis of certain brain tumors (e.g., pituitary tumors, nerve sheath tumors, and meningiomas), congenital anomalies (e.g., hydrocephalus), and traumatic lesions can be made with fairly high confidence based on characteristic imaging features. Similarly, even though histopathology is the gold standard in the diagnosis of inflammatory brain lesions, a presumptive diagnosis of meningoencephalomyelitis of undetermined etiology can be made based on a combination of signalment, neurological examination results, magnetic resonance imaging (MRI) findings, cerebrospinal fluid analysis, and negative infectious disease testing results (32). Nevertheless, it is possible that isolated cases in our patient cohort may have been misclassified. In a previous report, 24% of dogs with inflammatory CSF had a normal MRI study (33). It is theoretically possible that some cases of inflammatory disease may have been missed. We believe that this possibility is low, since at our institution patients with a normal brain MR examination almost always have CSF analysis performed as an ancillary diagnostic test. Similarly, even though cerebrovascular lesions may resemble intra-axial brain tumors, diffusion-weighted imaging (DWI), apparent diffusion coefficient (ADC) map, and T2*W GRE sequence are included in our standard MRI brain protocol, increasing confidence in the diagnosis of ischemic and hemorrhagic intra-cranial events (34). Another study limitation is that there was no requirement to have patient follow-up for a specific length of time. It is possible that the clinical diagnosis may have been different for some cases if extended follow-up had been available.

In previous studies that included histopathology, there may have been a bias for patients that had more severe disease and had to be euthanized or died. Without the inclusion criterion of histopathology, our study cohort was more representative of the spectrum of encephalopathies and of the caseload routinely imaged. Furthermore, since the categories were kept broad (i.e., radiologists did not have to specify the tumor type in the “neoplasia” category or the type of brain disease in the “other” category), this allowed for simplified categorization, decreased the number of variables, and helped keep the numbers in each category amenable to statistical analysis. In previous studies where radiologists were asked to interpret brain MRI studies in dogs, they may not have agreed with specific imaging features, however, they satisfactorily agreed on the category of brain disease (3).

Our study included both dogs and cats. It is difficult to draw specific conclusions for cats given their small number in our patient cohort. However, we felt that including all patients as they presented to the hospital for advanced imaging was a better representation of the natural population without bias.

Another possible source of bias may have been if the attending radiologist at the time of study interpretation would have had access to one of the MRI techniques under investigation (e.g., pre- and post-contrast T1W GRE images) but not the other (e.g., subtraction images), and that thus one of the two actors might have contributed more than the other to the MRI diagnosis and ultimately clinical diagnosis. We believe that this was not a significant factor in our study. Subtraction images were generated by the MRI technologist on the MRI scanning platform while the study was ongoing, and were sent to the reading platform (PACS) at the same time as the post contrast T1-W GRE images. The attending radiologist therefore had access to both and was free to base his/her interpretation on the study sequences and/or the standard pre- and post-contrast spin echo sequences also acquired in all cases.

One last limitation is that the two readers in our study were from the same institution where the MRI studies were originally acquired. A total of eight radiology residents and radiologists share the responsibility for clinical MRI case interpretation at our institution. It cannot be entirely excluded that there may have been isolated cases of re-call bias if a reader was asked to interpret an MRI study of a patient they may have previously seen while on clinical duty. However, we believe that this is negligible as the readers were blinded to patient identity, clinical findings, and other MRI sequences, as the images were randomized, and as there was a time gap of multiple months between presentation of a given patient to the hospital and the image evaluation.

Previous studies investigating the use of subtraction images in the evaluation of canine CNS disease were focused on inflammatory diseases and yielded conflicting results. One study found that subtraction images did not have advantages over T1W post contrast images and that, overall, MRI was poor at detecting meningeal pathology (23). Another study comparing subtraction images vs. pre- and post-gadolinium T1W spin echo (SE) image pairs concluded that the subtraction images performed better than the pre- and post-contrast imaging pairs (24). A possible explanation is that fat within the calvarium is hyperintense on standard SE sequences, which may mimic or obscure adjacent contrast enhancement. Subtraction imaging not only highlights contrast enhancing tissues in these cases, it also results in effective suppression of non-contrast-enhancing fat similar to the effect of chemical fat saturation. Chemical fat suppression resulted in significantly increased detection of meningeal enhancement in a previous study in dogs (26). The GRE sequence used in our study already included fat suppression, possibly negating this positive effect of subtraction images. One study evaluating the utility of MRI in the assessment of dogs with steroid responsive meningitis arteritis found improved depiction of meningeal contrast enhancement in the spine when using subtraction techniques, attributed to effective suppression of non-contrast-enhancing spinal epidural fat similar to the effect of chemical fat saturation (21). The same study found increased conspicuity of contrast enhancement associated with the articular facet joints and paraspinal musculature in some cases. Our study was focused on intracranial pathology only, and, unlike prior studies, included non-inflammatory encephalopathies. MRI subtraction images did not provide an advantage to paired pre- and post-contrast FS GRE images. Misinterpretation of “other” encephalopathies as “normal” is unlikely to represent a clinical problem as those diseases are likely going to be identified on other MRI sequences. However, misinterpretation of physiologic meningeal enhancement as abnormal, yielding an erroneous diagnosis of meningitis and inflammatory brain disease, represents a clinical pitfall.

This study leaves room for future investigation. In clinical practice, presence or absence of contrast enhancement would likely not be assessed by choosing pre- and post-contrast images or subtraction images in isolation, but would most likely being done by evaluating both in conjunction with other sequences. Providing reviewers with pre- and post-contrast image pairs first, and with pre-and post-contrast image pairs along with subtraction images later, may provide interesting insight if subtraction imaging provides meaningful additional information to the interpretation of T1W images alone.

## 5 Conclusion

Based on this study, subtraction images do not provide an advantage to paired pre- and post-contrast T1W FS GRE images when evaluating the canine and feline brain. The study results may not apply to instances where fat suppressed images are not available, when evaluating for concurrent extracranial abnormalities (e.g., muscle lesions), and when evaluating the canine and feline spine.

## Data availability statement

The raw data supporting the conclusions of this article will be made available by the authors, without undue reservation.

## Ethics statement

Ethical approval was not required for the studies involving animals in accordance with the local legislation and institutional requirements because this was a retrospective study involving image evaluation of MRI studies performed in the frame of the diagnostic work-up of small animal patients. Ethical review and approval is not required for a retrospective study at our institution. Written informed consent was obtained from the owners for the participation of their animals in this study.

## Author contributions

HS: Investigation, Data curation, Writing – original draft. SH: Investigation, Conceptualization, Methodology, Project administration, Resources, Supervision, Visualization, Writing – review & editing. CF: Investigation, Writing – review & editing. XS: Formal analysis, Investigation, Visualization, Writing – review & editing.
