# Supplementary material for: Magnetic resonance imaging subtraction vs. pre- and post-contrast 3D gradient recalled echo fat suppressed imaging for evaluation of the canine and feline brain
Source: Front Vet Sci. 2024 Jan 23;11:1346617. doi: 10.3389/fvets.2024.1346617 (PMC10844400; doi:10.3389/fvets.2024.1346617)

*Supplementary Material*

**Supplementary Figure 1.** Schematic representation of the percent agreement between the MRI Diagnosis based on paired pre- and post-contrast T1W FS GRE and the gold standard clinical diagnosis for reader 1.

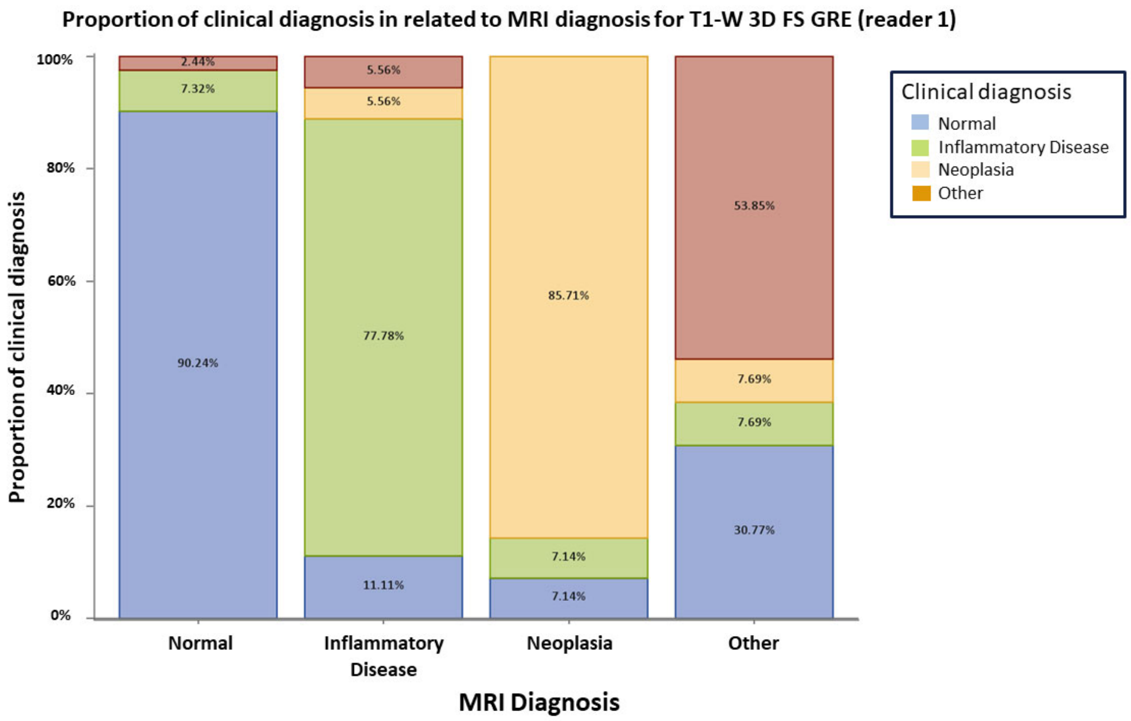

**Supplementary Figure 2.** Schematic representation of the percent agreement between the MRI Diagnosis based on paired pre- and post-contrast T1W FS GRE and the gold standard clinical diagnosis for reader 2.

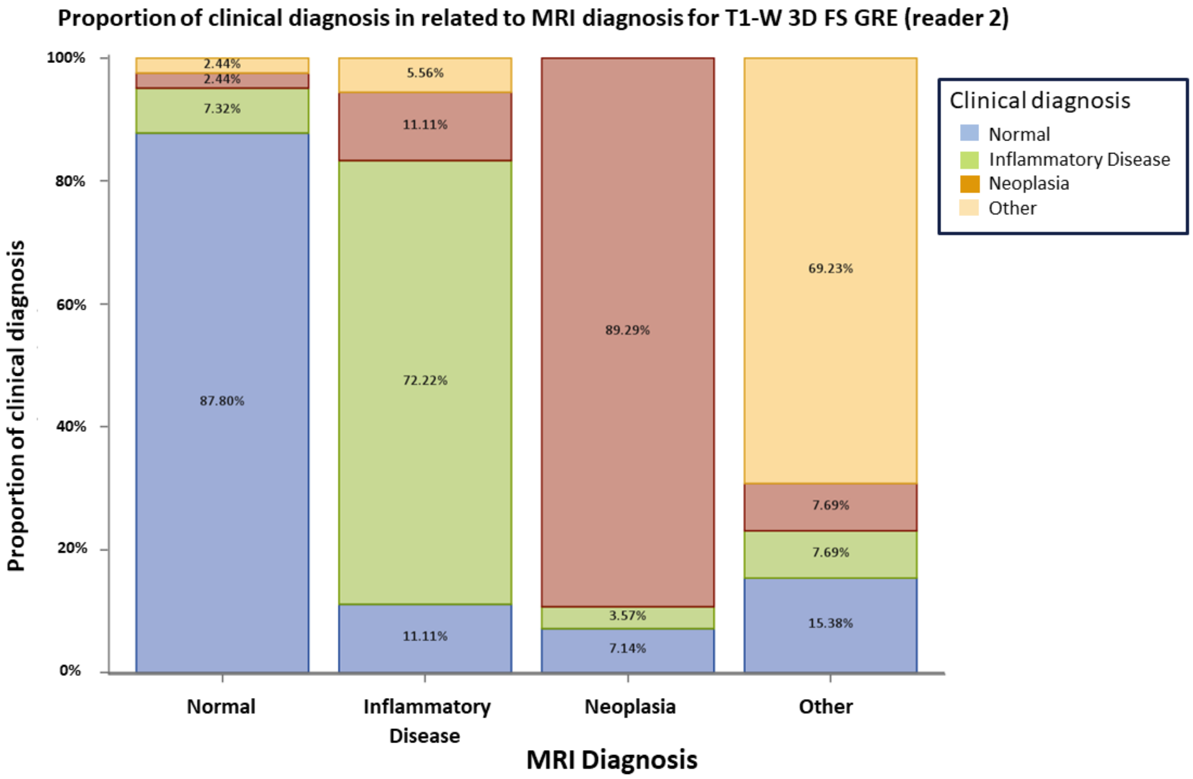

**Supplementary Figure 3.** Schematic representation of the percent agreement between the MRI Diagnosis based on subtraction MR image and the gold standard clinical diagnosis for reader 1.

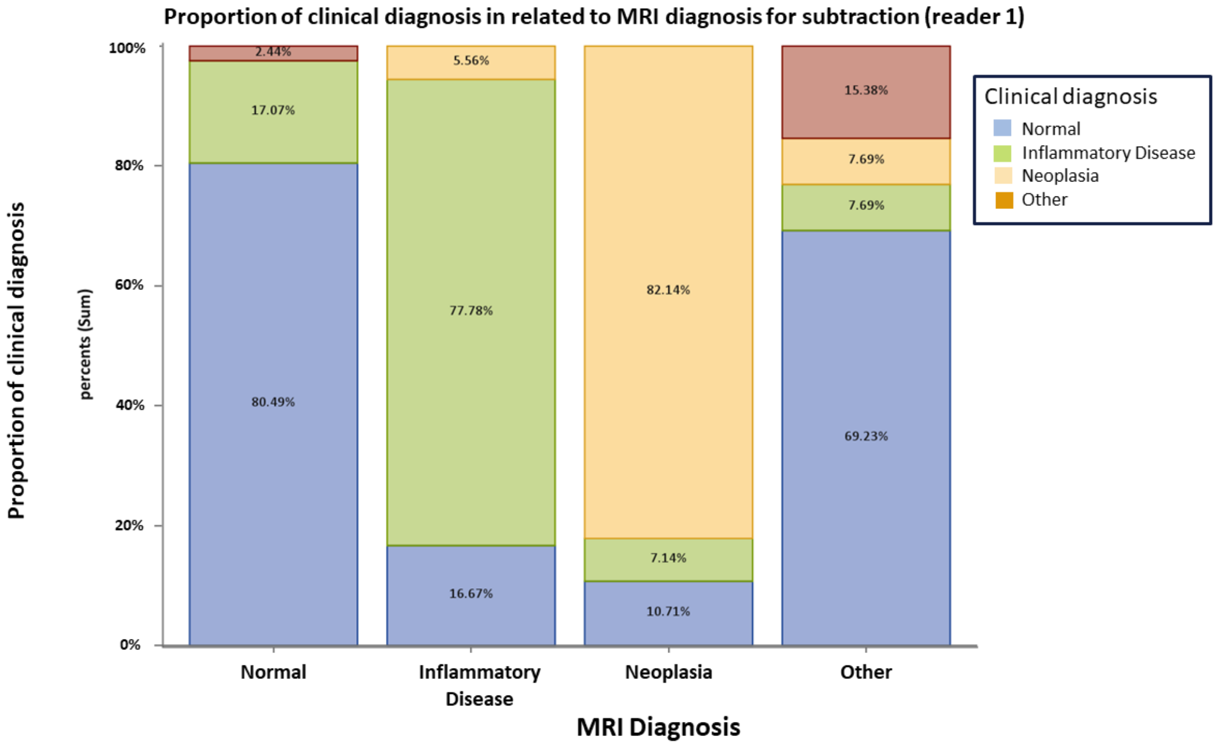

**Supplementary Figure 4.** Schematic representation of the percent agreement between the MRI Diagnosis based on subtraction MR image and the gold standard clinical diagnosis for reader 2.

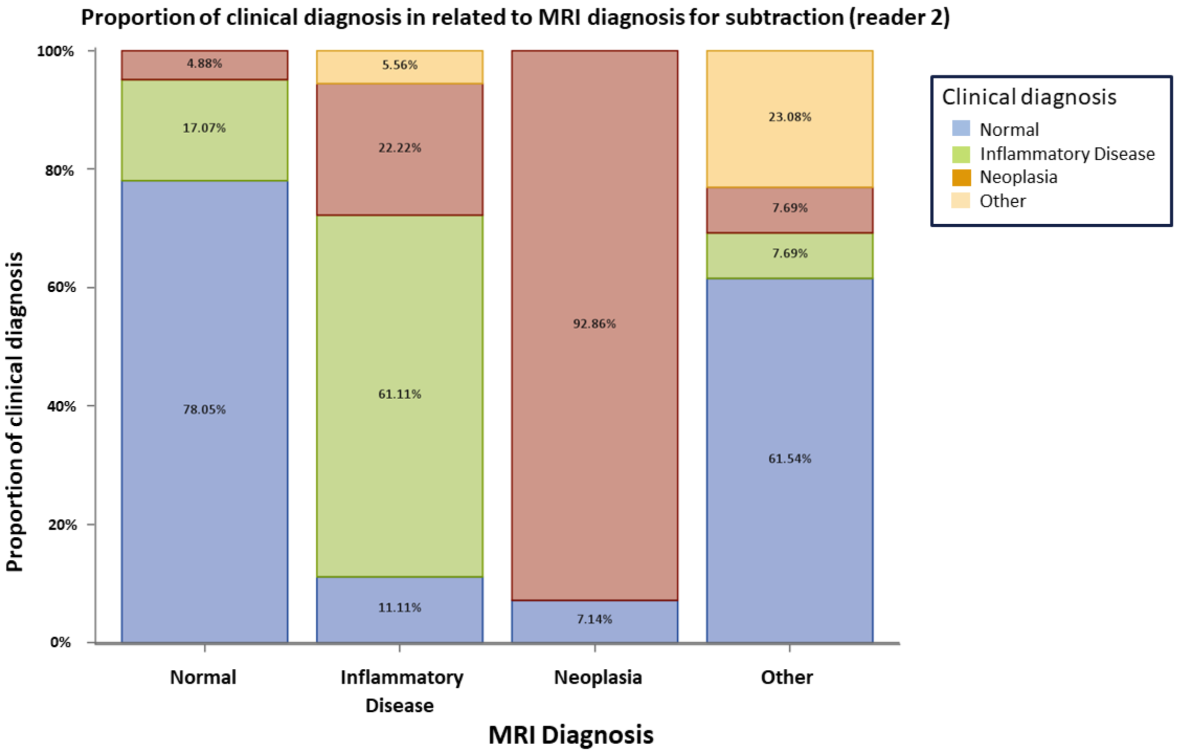

Supplement: Supplementary file 1 [file Data_Sheet_1.PDF]
